# Trachoma Control as a Vehicle Toward International Development and Achievement of the Millennium Development Goals

**DOI:** 10.1371/journal.pntd.0003137

**Published:** 2014-09-18

**Authors:** Kelly Callahan, Yasmin P. Ogale, Stephanie L. Palmer, Paul M. Emerson, Donald R. Hopkins, P. Craig Withers, Jeremiah M. Ngondi

**Affiliations:** 1 The Carter Center, Atlanta, Georgia, United States of America; 2 International Trachoma Initiative, Atlanta, Georgia, United States of America; 3 Department of Public Health and Primary Care, University of Cambridge, Cambridge, United Kingdom; 4 RTI International, Dar es Salaam, Tanzania; London School of Hygiene&Tropical Medicine, United Kingdom

Caused by bacterial infection, trachoma is the leading infectious cause of blindness in the world and is currently endemic in approximately 53 countries. Estimates report that 2.2 million people are visually impaired as a result of trachoma, 1.2 million of whom are irreversibly blind, and another 7.3 million persons are living with the late, disabling stages of the disease [1]. Trachoma is exacerbated by poverty, unsanitary living conditions, crowding, and the eye-seeking fly *Musca sorbens*. The World Health Organization (WHO) has endorsed the SAFE strategy for trachoma control, which stands for surgery, antibiotics, facial cleanliness, and environmental changes. The latest reports indicate that at least 110 million people live in areas where trachoma is suspected to be endemic and implementation of SAFE is necessary [2].

In the late 1990s, member states of the World Health Assembly passed a resolution calling for the elimination of blinding trachoma as a public health problem by 2020 and established the Global Alliance for the Elimination of Trachoma by 2020 (GET2020) to support this goal. Since then, considerable efforts have been made at both the international and national levels to control and reduce trachoma transmission and prevalence [1].

In light of these recent efforts, experience and anecdotal evidence gathered by leaders in the field suggest that the interventions employed as part of the SAFE strategy might work beyond trachoma-specific end goals; specifically, the basic interventions provided through SAFE can foster development and reduce poverty at the household and community levels. Intuitively, the conjecture makes sense: reductions in disease burden should work to increase productivity and income, while improvements in water sanitation and hygiene (WASH) increase quality of life and improve overall health. Taken together, the interventions could break the cycle of poverty and stimulate development.

Evidence from the literature in support of this claim (using the goals outlined by the United Nations [UN] Millennium Development Goals [MDGs] as a metric for development) is slim at best. While publications documenting the effectiveness of trachoma control in reducing trachoma burden are plentiful, studies measuring the impact of SAFE on development are less robust. A review of the present evidence base does prove to be hypothesis-generating, however. Analyzing each goal separately, there is a rational basis to support the argument that trachoma control works to improve development.

## MDG 1 (Eradicate Extreme Poverty and Hunger)

Trachoma exacerbates the poverty cycle with the additional burden of blindness and low vision [3]. Studies have quantified this impact, suggesting a total loss in productivity in the order of US$3.5 billion to US$5.3 billion per year (expressed in 2003 US$) [4]. Prevention of blindness via surgery, along with reduction in trachoma burdens through antibiotic treatment and sanitation improvements, reduces disability and could improve productivity at the individual and community levels.

## MDG 2 (Achieve Universal Primary Education)

Thus far, no studies have demonstrated an impact of SAFE on educational achievement or school enrollment. However, anecdotal evidence from trachoma control programs suggests that trachoma negatively impacts school attendance, particularly for the girls. In situations in which elders are affected by the disease, girls are usually pulled from school in order to assist with outstanding responsibilities in the home [5]. Should this be commonplace, reduction of trachoma could prevent this practice.

## MDG 3 (Promote Gender Equality and Empower Women)

Studies have shown women to be at two- to four-times higher risk of trachoma infection than men, due to their roles as mothers and caretakers (exposing them to children, who are known reservoirs of infection) [5]. Increased risk of infection can translate to increased disease burden, a corollary of which is decreased participation in income-generating activities and productivity due to disability and/or blindness. SAFE programs that target women have the potential to address inequities that result from differential disease burdens by gender.

## MDG 4 (Reduce Child Mortality)

In the trachoma-endemic setting of Ethiopia, mass drug administration with the antibiotic azithromycin has been associated with reduced odds of childhood mortality [6]. Azithromycin is also effective in the pediatric treatment of pneumonia and the reduction of diarrhea, both leading causes of death in children [7], [8]. Improved sanitation also significantly reduces morbidities associated with other diseases such as diarrhea and intestinal worms, having implications for better child survival [9].

## MDG 5 (Improve Maternal Health)

An estimated 15% of all maternal deaths are caused by infections in the first six weeks after childbirth, mainly as a result of unhygienic practices and poor infection control during labor and delivery [10]. Improvements in water and sanitation through SAFE's “F” and “E” components can result in spillover benefits impacting maternal health outcomes. Such improvements could also reduce burdens of disease associated with helminth infections, the negative consequences of which (anemia, poor pregnancy outcomes) pregnant women are particularly vulnerable to [11].

## MDG 6 (Combat HIV/AIDS, Malaria, and Other Diseases)

Beyond the fact that SAFE addresses this goal directly through the reduction of trachoma, administration of azithromycin has independently been shown to reduce and prevent malaria [12]. Azithromycin is also effective in treating several sexually transmitted infections (STIs) including: genital *Chlamydia trachomatis*, gonorrhea, chancroid, early developments of syphilis, and potentially other infections that may be present in conjunction with syphilis [13], [14]. Evidence indicates the sexual transmission of human immunodeficiency virus (HIV) might be enhanced in the presence of certain STIs [15]. Put together, it can be postulated that community-level administration of azithromycin for the treatment of trachoma could address burdens related to malaria and/or HIV in regions where coinfection occurs. Increased sensitivity to such outcomes as well as further research in disease pathologies is needed to ascertain this inference. In addition, improvements in water and sanitation (“F” & “E” components of SAFE) produce collateral benefits on other neglected tropical diseases such as soil transmitted helminths and schistosomiasis [16],[17].

## MDG 7 (Ensuring Environmental Sustainability)

The construction of pit latrines and promotion of hand-washing is integral to sustained trachoma control. Reports from trachoma control programs such as that in the Amhara Regional State of Ethiopia have shown increases in latrine coverage and household latrine ownership, which are sustained due to community demand for latrines [18]. Continual data collection on number of latrines constructed and monitoring of their usage is necessary to quantify SAFE's contribution to this goal.

## MDG 8 (Develop a Global Partnership for Development)

As previously mentioned, the mission of the GET2020 Alliance is to develop international partnerships and mobilize resources across sectors to aid in the goal of trachoma elimination by the year 2020 [1]. An example of this is the pharmaceutical company Pfizer Inc., which is currently donating uncapped quantities of the antibiotic azithromycin (Zithromax) toward trachoma control efforts. To date, Pfizer has donated over 340 million doses of Zithromax, reaching millions of people in a total of 28 countries (International Trachoma Initiative; http://trachoma.org/blog/2013).

## Conclusion

In reviewing the links between each MDG and SAFE, those links certainly seem to suggest that the SAFE strategy can work to reduce poverty and improve development beyond trachoma-specific outcomes in communities where it is implemented. Currently, however, the connections between trachoma control and development are no more than hypotheses. Further evidence and studies are needed to adequately measure, monitor, and evaluate such impacts.

As trachoma control programs continue and the 2020 goal of eliminating blinding trachoma nears, it is vital that national and regional trachoma control programs be cognizant of such non-trachoma related outcomes. Further study into potential ancillary relationships is strongly encouraged so that control programs and policies may be strengthened for maximal effect across populations. In an era where major donors work in terms of the MDGs and other development indicators, clear evidence of the impact of SAFE on community development and poverty reduction is a critical step toward increased funding of trachoma control programs, as well as the advocacy for the prevention, control, and eventual elimination of neglected tropical diseases at large.
